# Increased Growth Differentiation Factor 15 in Patients with Hypoleptinemia-Associated Lipodystrophy

**DOI:** 10.3390/ijms21197214

**Published:** 2020-09-29

**Authors:** Susan Kralisch, Annett Hoffmann, Juliane Estrada-Kunz, Michael Stumvoll, Mathias Fasshauer, Anke Tönjes, Konstanze Miehle

**Affiliations:** 1 Medical Department—Endocrinology, Nephrology, Rheumatology, University of Leipzig, 04103 Leipzig, Germany; susan.kralisch@medizin.uni-leipzig.de (S.K.); annett.hoffmann@medizin.uni-leipzig.de (A.H.); Juliane.Estrada-Kunz@medizin.uni-leipzig.de (J.E.-K.); michael.stumvoll@medizin.uni-leipzig.de (M.S.); mathias.fasshauer@ernaehrung.uni-giessen.de (M.F.); anke.toenjes@medizin.uni-leipzg.de (A.T.); 2 IFB AdiposityDiseases, Leipzig University Medical Center, University of Leipzig, 04103 Leipzig, Germany; 3 Department of Nutritional Sciences, University of Giessen, 35390 Gieβen, Germany

**Keywords:** adipokine, GDF15, insulin resistance, leptin, lipodystrophy, obesity, triglycerides

## Abstract

**Objective**. Similar to obesity, lipodystrophy (LD) causes adipose tissue dysfunction and severe metabolic complications. Growth differentiation factor 15 (GDF15) belongs to the transforming growth factor β superfamily and is dysregulated in metabolic disease including obesity and diabetes mellitus. Circulating levels in LD and the impact of leptin treatment have not been investigated so far. **Material and Methods**. GDF15 serum levels were quantified in 60 LD patients without human immunodeficiency virus infection and 60 controls matched for age, gender, and body mass index. The impact of metreleptin treatment on circulating GDF15 was assessed in a subgroup of patients. GDF15 mRNA expression was determined in metabolic tissues of leptin-deficient lipodystrophic aP2-nSREBP1c-Tg mice, obese *ob/ob* mice, and control C57Bl6 mice. **Results**. Median GDF15 serum concentrations were significantly higher in LD patients (819 ng/L) as compared to the control group (415 ng/L) (*p* < 0.001). In multiple linear regression analysis, an independent and positive association remained between GDF15 on one hand and age, patient group, hemoglobin A1c, triglycerides, and C-reactive protein on the other hand. Moreover, there was an independent negative association between GFD15 and estimated glomerular filtration rate. Circulating GDF15 was not significantly affected by metreleptin treatment in LD patients. *Gdf15* was upregulated in leptin-deficient lipodystrophic mice as compared to controls. Moreover, *Gdf15* mRNA expression was downregulated by leptin treatment in lipodystrophic and obese animals. **Conclusions**. Serum concentrations of GDF15 are elevated in LD patients and independently associated with markers of metabolic dysfunction. *Gdf15* expression is higher in lipodystrophic mice and downregulated by leptin treatment.

## 1. Introduction

Adipocyte hypertrophy and hyperplasia in obesity causes adipose tissue (AT) dysfunction and severe metabolic complications including dyslipidemia, nonalcoholic fatty liver disease, insulin resistance, diabetes mellitus, and coronary heart disease [1]. Paradoxically, the loss of AT in lipodystrophy (LD) syndromes is associated with the same complications [2]. LD syndromes are diseases of heterogeneous etiopathology (congenital or acquired) sharing the symptoms of selective reduction and dysfunctionality of subcutaneous adipose tissue (sAT) in large body sections [2]. Thereby, AT in LD has limited storage capacity for energy in terms of triacylglycerols. This leads to ectopic fat deposition predominantly in liver, pancreas and muscle [3]. In addition—and similar to obesity—AT paucity causes dysregulation of several endocrine adipocyte-derived factors, e.g., leptin and adiponectin, which are critical for the adequate regulation of glucose metabolism and energy homeostasis [4,5]. Supplementation of leptin resulted in significant improvements of glucose and lipid metabolism in LD animal models and in LD affected humans [6,7]. However, leptin treatment effects in humans vary substantially, especially in patients with partial LD [8]. Thus, additional treatment options are needed.

Growth differentiation factor 15 (GDF15), also known as non-steroidal anti-inflammatory drugs activated gene 1 or macrophage inhibitory cytokine-1, is a member of the transforming growth factor β superfamily [9]. Originally, it was described as a protein secreted by activated macrophages [9]. Further studies support the hypothesis, that GDF15 is more than a general stress induced cytokine. It regulates food intake, energy expenditure, and body weight in response to metabolic and toxin-induced stresses [10,11,12,13,14]. Only a limited number of tissues express GDF15 under physiological conditions, predominantly human placenta and—in markedly smaller amounts—other tissues, including colon, prostate, and kidney [15,16,17]. However, it is present in the circulation of healthy individuals in concentrations unusually high for a cytokine and is upregulated in a broad range of disease states, e.g., anorexia, cancer, inflammatory, and cardiovascular disease [18,19,20,21]. In obesity and diabetes mellitus, GDF15 upregulation has been described as well [22,23]. Moreover, GDF15 plays a role in regulation of energy balance, body weight, and fat mass [24,25].

Regulation of GDF15 has not been analyzed in patients with LD until now. In this study, we measured serum concentration of GDF15 in patients with acquired and congenital LD as compared to a healthy control group matched for age, body mass index (BMI), and gender. We assessed associations between circulating GDF15 and anthropometric and metabolic parameters. To reveal a possible impact of metreleptin treatment on GDF15 concentration, we determined GDF15 serum levels in 16 LD patients scheduled for metreleptin supplementation before and after 6 months of metreleptin therapy. Animal studies were performed to analyze the gene expression of *Gdf15* in different tissues and to analyze the influence of leptin treatment on the transcriptional regulation of GDF15.

## 2. Results

### 2.1. LD Patients Have a Significantly Impaired Metabolic Profile and Higher GDF15 Serum Concentrations than Controls

Anthropometric as well as laboratory parameters of LD patients and the control cohort are shown in Table 1. Patients with LD have significantly higher median GDF15 serum concentrations (819 ng/L) than non-LD controls (415 ng/L; *p* < 0.001) (Table 1). These results remain significant after exclusion of the metformin treated LD patients from the analysis (807 ng/L vs. 500 ng/L; *p* < 0.001).

Waist-to-hip ratio (WHR) and systolic blood pressure (SBP) are higher in the LD group as compared to controls (*p* < 0.001; Table 1). Moreover, patients with LD show significantly impaired parameters of glucose control (i.e., increased glycosylated hemoglobin A1c [HbA1c], fasting glucose [FG], fasting insulin [FI], and homeostasis model assessment of insulin resistance [HOMA-IR]) and lipid metabolism (i.e., decreased high density lipoprotein [HDL] cholesterol, increased triglycerides [TG], increased free fatty acids [FFA]) as compared to the control cohort (*p* < 0.05; Table 1). Interestingly, low density lipoprotein [LDL] cholesterol is significantly lower in LD patients than in controls (*p* < 0.001; Table 1). Additionally, patients with LD have a significantly better kidney function (i.e., lower creatinine [*p* = 0.011] and higher estimated glomerular filtration rate (eGFR) [*p* = 0.043]). C reactive protein (CRP) is slightly but significantly higher in the LD group as compared to controls (1.7 mg/L vs. 0.7 mg/L; *p* = 0.016; Table 1). Adiponectin and leptin serum levels are significantly lower in LD patients than in controls (*p* < 0.001; Table 1), while serum concentrations of fibroblast growth factor (FGF) 21 are significantly elevated in the LD group compared to controls (*p* = 0.002; Table 1). Serum markers of liver functionality, i.e., alanine aminotransferase (ALAT), aspartate aminotransferase (ASAT), and gamma-glutamyl transferase (GGT) are significantly upregulated in LD patients as compared to controls (Table 1).

### 2.2. Univariate and Multivariate Analyses

When analyzing the entire study cohort, circulating GDF15 serum levels are positively associated with age, BMI, WHR, SBP, HbA1c, FG, FI, HOMA-IR, TG, CRP, FGF-21, smoking, and metformin intake (*p* < 0.05; Table 2). A significant and negative correlation is found between GDF15 levels and HDL cholesterol, LDL cholesterol, eGFR, adiponectin, and leptin (*p* < 0.05; Table 2). There is no significant association of serum GDF15 concentration with diastolic blood pressure (DBP), cholesterol, FFA, and creatinine (Table 2).

In multiple linear regression analysis, an independent and positive association remains between GDF15 and age, group (LD vs. non-LD), HbA1c, TG, as well as CRP. Moreover, there is a negative association between GDF15 and eGFR (Table 2).

### 2.3. GDF15 Serum Levels during Metreleptin Supplementation

Most pronounced changes in anthropometric and metabolic parameters were found after 6 months of metreleptin supplementation. Thus, BMI (27.3 kg/m^2^ versus 27.4; *p* = 0.035) slightly but significantly decreased after 6 months as compared to baseline. Moreover, median HbA1c (7.2% versus 8.0%; *p* = 0.08) and TG levels (3.97 mmol/L versus 8.64 mmol/L; *p* = 0.020) decreased but only the latter reached statistical significance. Parameters of liver function, i.e., ALAT, ASAT, and GGT did not significantly change during 6 months of metreleptin treatment. As expected, leptin serum concentration increased during metreleptin treatment from 5.1 to 11.8 µg/L (*p* = 0.023).

There is no significant change in serum GDF15 levels between baseline and 6 months of metreleptin treatment in a subcohort of LD patients (Table 3).

### 2.4. GDF15 mRNA Expression in a Mouse Model of Congenital Generalized LD and Leptin-Deficient Obesity

The animal model of congenital generalized LD is characterized by loss of sAT and epididymal AT (epiAT), impaired lipid metabolism and liver function, and significantly improved by leptin treatment comparable to the human cohort (Table 4).

**Figure 1:**
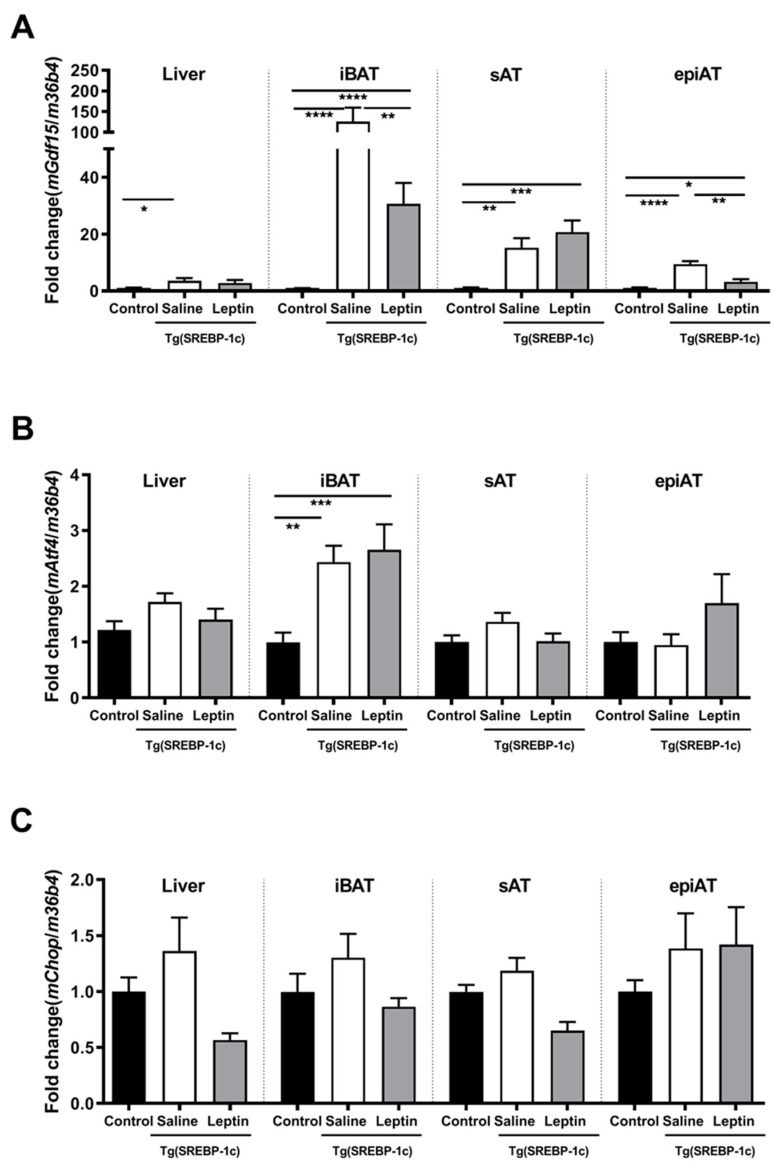
Effect of leptin treatment (3.0 mg/kg BW/d) on *Gdf15* (**A**), *Aft4* (**B**), and *Chop* (**C**), mRNA expression relative to *36B4* (*n* ≥ 8 per group) in liver, intrascapular brown (iBAT), subcutaneous (sAT), and epididymal (epiAT) adipose tissue of Tg(aP2-SREBF1c). Data are presented as means ± SEM. Differences were assessed by one-way ANOVA corrected by Bonferroni–Holm. * indicates *p* < 0.05, ** *p* < 0.01, *** *p* < 0.001, **** *p* < 0.0001.

**Figure 2:**
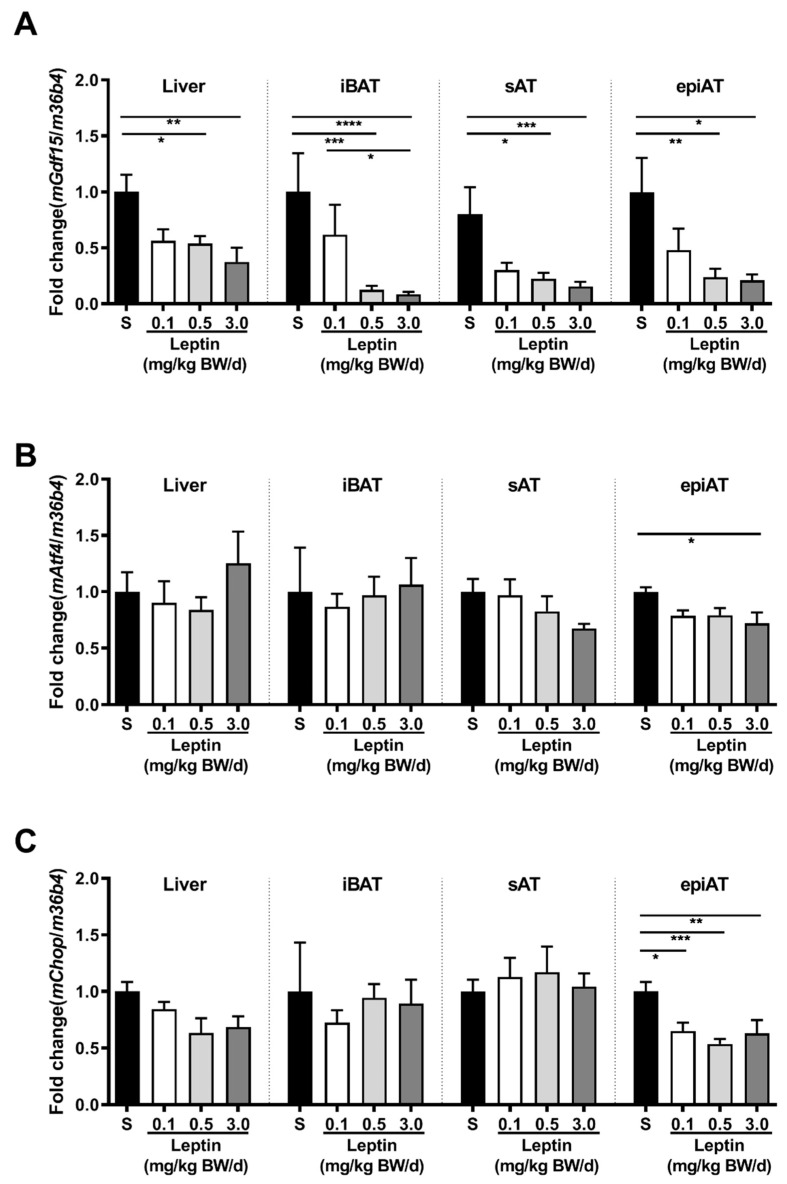
Effect of leptin treatment (0.1, 0.5, and 3.0 mg/kg BW/d) on *Gdf15* (**A**), *Aft4* (**B**), and *Chop* (**C**) mRNA expression relative to *36B4* (*n* ≥ 8 per group) in liver, intrascapular brown (iBAT), subcutaneous (sAT), and epididymal (epiAT) adipose tissue of LDLR-/-;*ob/ob* animals. Data are presented as means ± SEM. Differences were assessed by one-way ANOVA corrected by Bonferroni–Holm. * indicates *p* < 0.05, ** *p* < 0.01, *** *p* < 0.001, **** *p* < 0.0001.

In Tg(aP2-SREBP-1c), *Gdf15* mRNA expression is significantly higher in all analyzed tissues in saline-treated LD mice as compared to non-LD control animals (Figure 1A). Leptin treatment significantly reduces *Gdf15* in intrascapular brown AT (iBAT) and epiAT in LD mice (Figure 1A). In additional analyses, activating transcription factor (*Atf*)*4* and C/EBP homologous protein (*Chop*) are analyzed as important upstream transcription regulators of *Gdf15. Atf4* mRNA is significantly up-regulated in saline-treated LD mice as compared to non-LD control animals in iBAT, but increased expression is not reversed by leptin treatment (Figure 1B). In contrast, *Chop* mRNA expression does not show any significant differences between the three groups and tissues (Figure 1C). In obese *ob/ob* animals, *Gdf15* mRNA expression in liver, iBAT, sAT, and epiAT is dose-dependently decreased by leptin treatment (Figure 2A) as compared to saline-treated mice. This regulation is accompanied by decreased mRNA expression of *Atf4* and *Chop*, in epiAT (Figure 2B,C).

Interestingly, m*Gdf15* is significantly increased in sAT of lipodystrophic Tg(aP2-SREBP-1c) animals compared to *ob/ob* animals, while in liver Tg(ap2-SREBP-1c) mice show a significant decreased gene expression profile as compared to *ob/ob* animals. The gene expression of *Gdf15* in iBAT and epiAT is not significantly different in obese and lipodystrophic animals. *Chop* gene expression is significantly lower expressed in Tg(aP2-SREBP-1c) as in *ob/ob* animals in iBAT, while *Atf4* is not differentially expressed between these both animal models (data not shown).

## 3. Discussion

In the current study, we demonstrate for the first time that circulating GDF15 concentrations are significantly higher in LD patients as compared to age-, gender-, and BMI-matched controls.

Since it has been described that oral metformin exerts its effects on body weight and energy balance via GDF15 [26], we have reanalyzed our data after excluding metformin-treated LD patients. Interestingly, the difference in GDF15 serum levels between LD and controls remains significant, indicating a metformin independent mechanism for increased GDF15 serum levels in LD.

GDF15 concentrations are elevated in various diseases. Thus, GDF15 is associated with cardiovascular morbidity and mortality [27,28,29,30]. Moreover, in individuals with obesity or diabetes, circulating levels of GDF15 are higher than in unaffected individuals, and are positively correlated with serum glucose, HbA1c, and insulin resistance [22,31,32].

However, even after excluding all LD patients with diabetes mellitus from the calculation, GDF15 is still significantly elevated in the LD cohort (data not shown). Interestingly, in a cohort of 876 male subjects from the Swedish Population Registry, high GDF15 levels are predictive of all-cause mortality independent of age, BMI, and smoking [33]. These findings are confirmed by another longitudinal study among 1391 subjects without cardiovascular disease followed for at least 11 years [34]. Since LD patients are not only at risk for cardiovascular events and diabetes, but also have an increased morbidity and mortality due to other complications e.g., acute pancreatitis and liver failure [35], increased GDF15 levels might be a predictor for elevated overall morbidity and mortality in LD.

The pathophysiology of elevated GDF15 concentrations in patients with LD still has to be elucidated. Hence, GDF15 provides an endocrine signal of nutritional stress in mice and humans [36]. Since patients with LD suffer from loss of satiety caused by the lack of the adipose-tissue-derived leptin, permanent hunger and chronic overfeeding is common in LD [37], which might be one possible explanation for elevated GDF15 levels in LD. However, Tsai et al. [25] could not find a substantial postprandial increase in GDF15 serum concentration. Thus, GDF15, unlike leptin, is not acting as a satiety factor but as a regulator of energy homeostasis, most likely due to long-term effects [25].

Upregulation of *Gdf15* expression in various insulin-sensitive tissues of LD mice might be due to the metabolic challenges in LD. This hypothesis is supported by our leptin treatment studies in LD mice and genetically obese animals. In both animal models, *Gdf15* expression is significantly reduced after leptin treatment, which is possibly a consequence of metabolic improvement. Leptin dose-dependent regulation is most pronounced in epiAT of *ob/ob* animals emphasized by the down-regulation of *Gdf15*, as well as upstream transcriptional regulators *Atf4* and *Chop.* Leptin-induced weight loss in *ob/ob* animals might play a decisive role [38]. In accordance to our rodent data, markers for metabolic and vascular disease are independent predictors for circulating GDF15 in our human LD cohort. Thus, HbA1c, TG, and CRP are independently and positively associated with circulating GDF15, whereas eGFR is a negative independent predictor.

These findings are in accordance with numerous studies implicating GDF15 in a variety of age-related disorders, such as cardiovascular diseases and diabetes [22,33,39]. Published data in obese and type 2 diabetic woman show highest GDF15 serum levels in obese patients with type 2 diabetes as compared to lean women [31]. Insulin sensitivity (assessed by oral glucose sensitivity, HOMA-IR, and HbA1c) has been shown to be an independent predictor of GDF15 levels [22,40]. However, another study shows that baseline GDF15 levels are significantly higher in individuals who subsequently develop type 2 diabetes than in those who remain diabetes-free, but that these levels are not independently associated with the incidence of type 2 diabetes [41]. Moreover, recombinant GDF15 improves glucose and insulin tolerance in high fat diet mice but also (to a lesser extend) in chow-fed mice [42]. These findings suppose that GDF15 is not only a biomarker giving information on severity of metabolic homeostasis in LD, but also might be a compensatory mechanism to avoid LD-linked metabolic disorders. Further cohort and clinical studies will be necessary to confirm this hypothesis in LD.

CRP is an independent and positive predictor of GDF15 serum levels in our study, while in cohorts of incident dialysis patients from Sweden and US [43] and in patients selected from the Swedish Population Registry [33], it does not sustain multivariate analysis. Appropriately, in 2019, our workgroup published that leptin administration within the subphysiological to physiological range to *ob/ob* animals diminishes circulating pro-inflammatory interleukin-6 and macrophage chemoattractant protein-1, leading to decreased AT macrophage infiltration [44]. Accordingly, AT macrophages are even more abundant in LD than in obesity [45]. Further studies have to be performed to assess the clinical significance of current findings in leptin deficient *ob/ob* and LD animals.

Further on, LD is characterized by diminished adipose tissue and hypoleptinemia, leading to ectopic TG accumulation associated with liver steatosis, potentially leading to nonalcoholic fatty liver disease (NAFLD) [46]. Related to NAFLD are elevated TG levels, which we showed GDF15 is positively and independently associated with. Interestingly, in NAFLD, GDF15 may predict disease severity, advanced fibrosis, and cirrhosis [47,48]. The gene expression of *Gdf15* is increased in NAFLD livers of animal models and human subjects. GDF15-knockout mice exhibited aggravated NAFLD phenotypes such as increased steatosis, hepatic inflammation, fibrosis, liver injury, and metabolic deterioration [49] and expression of hepatic *Gdf15* reduced lipid accumulation in liver and NAFLD progression [50]. In accordance, GDF15-transgenic mice show attenuation of NAFLD phenotypes and metabolic deterioration [49]. Thus, GDF15 might exert beneficial functions in regulating lipid metabolism of NAFLD in LD. Clearly, further investigations using e.g., transient elastography should clarify a link between GDF15 and progression of fatty liver disease in our study cohort.

Elevated plasma levels of GDF15 have been linked to end-stage renal failure [43].

In our human study, GDF15 is also negatively associated with the glomerular filtration rate, which might be link to conspicuously frequent incidence of chronic kidney disease in patients with LD [51].

In contrast to our mouse experiments, where leptin treatment significantly reduces mRNA expression of *Gdf15* in iBAT, and epiAT in LD mice (Figure 1A), serum GDF15 concentration in humans did not change after a 6 month course of metreleptin treatment instead of improvement in metabolic parameters (Table 3). Similarly, GDF15 serum levels at 1 week, 1 month, 3 months, and 12 months of metreleptin treatment are not significantly different to baseline (data not shown). These discrepancies might be based on several reasons: Unlike the investigated mouse model that mimics congenital generalized LD, the majority of LD patients in our metreleptin treatment cohort (i.e., 14 out of 16 LD patients) suffer from partial LD. This has also to be taken in account when comparing the metabolic outcome of our metreleptin trial with the results of other metreleptin treatment studies that often enrolled much more patients with generalized LD. Two studies have been revealed that patients with generalized LD often show a more pronounced response to metreleptin supplementation as compared to patients with partial LD [8,52]. This finding has been confirmed in our treatment group (data not shown). Thus, in the two patients with generalized LD, glucose control and lipid metabolism have clearly improved during treatment with metreleptin. Moreover, GDF15 serum levels decreased in these patients (data not shown).

Taken together, elevation of GDF15 in LD is most likely a response to impaired glucose homeostasis, lipid metabolism, and liver function in LD and might be a protective mechanism for the body. This is supported by the study in transgenic mice overexpressing GDF15 and having high GDF15 serum concentrations who live much longer than their wild type counterparts irrespective of diet [53]. The impact of leptin on GDF15 regulation is significant in animals, but larger patient cohorts with a more homogenous patient population (e.g., including only patients with generalized LD) might be required to reveal a significant effect of metreleptin treatment on GDF15 serum levels in humans.

## 4. Material and Methods

### 4.1. Patients and Control Group

Sixty patients with non-human immunodeficiency virus-associated LD (12 male/48 female, age 16–74 years, BMI 16.8–33.5 kg/m^2^) gave their informed consent for participation in the study. In detail, the LD cohort consisted of 53 patients with familial partial LD (mutation in *Lamin A/C n* = 30; mutation in peroxisome proliferator-activated receptor gamma *n* = 7; no LD specific mutation but suspected genetic origin due to positive family history *n* = 16), 2 patients with acquired partial LD, 2 patients with congenital generalized LD (mutation in *DNA polymerase delta1 n* = 1; mutation in polymerase I and transcript release factor *n* = 1), and 3 patients with acquired generalized LD. Diagnosis was made according to Brown et al. [35] by clinical phenotype as well as genetic testing in case of inherited forms. Twenty-eight of the 60 LD patients were treated with metformin for diabetes mellitus. The control group comprised 60 healthy subjects without LD or metabolic disease (e.g., diabetes mellitus, hypertriglyceridemia). Patients and controls were matched for age, gender, and BMI. Patients and controls were recruited from the Leipzig Lipodystrophy Centre at the Division of Endocrinology of the University of Leipzig.

Sixteen of the 60 LD patients (13 female/3 male; 14 partial LD/2 generalized LD) met the inclusion criteria for metreleptin treatment. Inclusion criteria were confirmed LD, insufficiently controlled diabetes mellitus and/or hypertriglyceridemia despite adequate antihyperglycemic and/or lipid-lowering treatment, age ≥ 5 years at baseline, and eGFR > 40 mL/min. Exclusion criteria included HIV infection infectious liver disease, primary hematologic abnormalities, active malignant tumor, pregnancy or lactation, and hypersensitivity to E. coli-derived proteins. Metreleptin was provided by the manufacturer (i.e., Amylin [San Diego, CA, USA]/Bristol-Myers-Squibb [Munich, Germany]/Astra Zeneca [London, UK]/Aegerion [Cambridge, MA, USA], respectively within the scope (framework) of a compassionate use program. The first three patients (all female) administered metreleptin subcutaneously for the first week at 0.04 mg/kg body weight and thereafter at 0.08 mg/kg body weight. Since dosing instructions changed, patients 4 to 16 received 2.5 mg metreleptin per day (men) and 5 mg metreleptin per day (women). Metreleptin doses from second week onwards ranged from 2.5 to 7.8 mg per day. [54]. Lipid-lowering and antihyperglycemic medication was modified if necessary. Comprehensive anthropometric and metabolic exploration was done in all 16 patients at several times during the treatment (1 week, and 1, 3, 6, and 12 months, respectively). In view of the fact that metreleptin’s glucose- and lipid-lowering power was most effective at 6 months of treatment, these data were used for calculation. The study was approved by the Leipzig University Ethics Committee (135/13-ek, 08/07/2013).

### 4.2. Anthropometric Measurements and Laboratory Assessment

We calculated HOMA-IR and eGFR according to Matthew et al. [55] and Levey et al. [56], respectively. BMI was estimated as weight (in kilogram) divided by squared height (in meters). Evaluation of WHR was performed after measuring waist and hip circumferences.

In all participants, blood samples were taken in the morning after a fasting period of 8 h or longer. For measurements of adipokine serum concentrations commercially available enzyme-linked immunosorbent assays from Mediagnost (Reutlingen, Germany) for leptin and adiponectin from Biovendor (Modrice, Czech Republic) for fibroblast growth factor (FGF) 21, and from R&D Systems (Minneapolis, MN, USA) for GDF15 were used according to the manufacturers’ instructions. Routine laboratory parameters of glucose homeostasis, (i.e., FI, FG, and HbA1c), lipid metabolism, (i.e., total, HDL, and LDL cholesterol, FFA, and TG, kidney function, (i.e., creatinine), inflammation (i.e., CRP), and liver function (i.e., ALAT, ASAT, GGT) were assessed by standard laboratory methods in a certified laboratory.

### 4.3. Animal Care and Animal Experiments

Mouse breeding and experiments were performed in the Medical Experimental Center, University of Leipzig in compliance with animal welfare regulations. Animal protocols were approved by the local ethics committee (approval no. TVV37/12, 06/11/2012 and TVV 27/16, 22/09/2016). All mice were on a C57Bl/6 and low-density lipoprotein receptor knockout (LDLR−/−) background. They were maintained in a room under pathogen-free conditions with controlled 21 ± 1 °C on a 12:12 h light/dark cycle (6 a.m./6 p.m.). Eight-weeks-old male Tg(aP2-SREBF1c) mice, a transgenic murine model for congenital generalized lipodystrophy [57], or *ob/ob* mice were randomized into two and four groups, respectively. The groups were treated daily i.p. with recombinant leptin (Tg(aP2-SREBP1c): 3.0 mg/kg BW and *ob/ob*: 0.1 mg/kg body weight [BW], 0.5 mg/kg BW, and 3.0 mg/kg BW; R&D Systems, Wiesbaden-Nordenstadt, Germany) or saline for 8 weeks. At 3.0 mg/kg BW/d, leptin has physiological effects, i.e., this dose is sufficient to normalize BW in leptin-deficient *ob/ob* mice (data not shown). Non-LD littermates on a LDLR−/− background served as controls for Tg(aP2-SREBF1c) mice. At the end of the treatment period, the mice were sacrificed and iBAT, epiAT, sAT, and liver were weighted and snap frozen.

For metabolic characterization, plasma glucose, cholesterol, TG, FFA, ALAT, and ASAT were analyzed by standard laboratory methods in a certified laboratory.

### 4.4. Quantitative Real-Time RT-PCR Analysis

Quantitative real-time RT-PCR for determination of *Gdf15* mRNA synthesis relative to *36B4* has been performed using the following mouse primers: *Gdf15*: 5′-CAAGTCCTGACCCAGCTGTC-3′, and 5′-TCAGGGGCCTAGTGATGTCC-3′; *36B4*: 5′-AAGCGCGTCCTGGCATTGTCT-3′, and 5′-CCGCAGGGGCAGCAGTGGT-3′, *Atf*4: 5′-GGGTTCTGTCTTCCACTCCA-3′, and 5′-AAGCAGCAGAGTCAGGCTTTC-3′ and (*Chop*) 5′-CCACCACACCTGAAAGCAGAA-3′, and 5′-AGGTGAAAGGCAGGGACTCA-3′ (forward and reverse, respectively).

### 4.5. Statistical Analysis

We used SPSS Statistics Version 24.0 (IBM, Armonk, NY, USA) for all statistical analyses in humans and GraphPad Prism 6 (GraphPad Software, San Diego, CA, USA) for all animal data sets. The non-parametric Mann–Whitney U test was applied to reveal differences between the LD and the control cohort. Univariate correlations were determined by Spearman’s rank correlation test. We performed multivariate linear regression analysis for identification of independent relationships after testing parameters for normal Gaussian distribution by Shapiro–Wilk W test as well as logarithmic transformation of all non-normally distributed parameters. For the evaluation of differences in metabolic parameters and GDF15 serum levels before and during the course of metreleptin treatment, we used the non-parametric Wilcoxon signed rank test. Differences in animal models were assessed by one-way ANOVA corrected by Bonferroni–Holm after testing parameters for normal Gaussian distribution by Shapiro–Wilk W test as well as logarithmic transformation of all non-normally distributed parameters. All data are presented as median and interquartile range for human and as means ± standard error of the mean for mouse studies. *p*-values of < 0.05 were considered as statistically significant.

## Figures and Tables

**Table 1:** Baseline characteristics of the entire study population (*n* = 60 controls and *n* = 60 LD).

Parameter	Controls	LD	*p*
*n*	60	60	
GDF15 (ng/L)	414.9 (257.6)	818.9 (881.6)	<0.001 *
Age (years)	39 (22)	42 (24)	0.591
Gender (male/female)	12/48	12/48	-
BMI (kg/m^2^)	24.6 (4.9)	25.2 (4.6)	0.193
WHR	0.81 (0.11)	0.97 (0.11)	<0.001 *
SBP (mmHg)	122 (22)	131 (19)	<0.001 *
DBP (mmHg)	78 (15)	81 (14)	0.183
HbA1c (%)	5.2 (0.6)	6.0 (2.1)	<0.001 *
HbA1c (mmol/mol)	33.3 (6.3)	42.4 (23.0)	<0.001 *
FG (mmol/L)	5.2 (0.8)	5.6 (3.8)	0.020 *
FI (pmol/L)	51.8 (45.8)	114.9 (113.6)	<0.001 *
HOMA-IR	1.7 (1.7)	4.9 (5.8)	<0.001 *
Cholesterol (mmol/L)	5.36 (1.35)	5.29 (2.05)	0.258
HDL cholesterol (mmol/L)	1.54 (0.59)	0.85 (0.52)	<0.001 *
LDL cholesterol (mmol/L)	3.56 (1.39)	2.74 (1.76)	<0.001 *
TG (mmol/L)	0.98 (0.60)	2.92 (5.82)	<0.001 *
FFA (mmol/L)	0.44 (0.21)	0.61 (0.28)	0.002 *
Creatinine (µmol/L)	76 (20)	67 (21)	0.011 *
eGFR (mL/min/1.73 m^2^)	94.0 (19.0)	100.2 (31.7)	0.043 *
CRP (mg/L)	0.7 (1.5)	1.7 (2.5)	0.016 *
Adiponectin (mg/L)	9.3 (7.7)	2.7 (3.7)	<0.001 *
Leptin (µg/L)	12.0 (13.9)	4.3 (4.7)	<0.001 *
FGF21 (pg/mL) ^#^	184.4 (236.8)	381.8 (530.0)	0.002 *
Smoking (*n*)	7/60	18/59	0.014 *
Metformin (*n*)	0/60	28/60	<0.001 *
ALAT (µkat/L)	0.34 (0.20)	0.49 (0.42)	<0.001 *
ASAT (µkat/L)	0.33 (0.08)	0.48 (0.28)	<0.001 *
GGT (µkat/L)	0.28 (0.20)	0.65 (0.60)	<0.001 *

ALAT, alanine aminotransferase; ASAT, aspartate aminotransferase; BMI, body mass-index; CRP, C reactive protein; DBP, diastolic blood pressure; eGFR, estimated glomerular filtration rate; FFA, free fatty acids; FG, fasting glucose; FGF21, fibroblast growth factor 21; FI, fasting insulin; GDF15, growth differentiation factor 15; GGT, gamma-glutamyl transferase; HbA1c, glycosylated hemoglobin A1c; HDL, high-density lipoprotein; HOMA-IR, homeostasis model assessment of insulin resistance; LD, lipodystrophy; LDL, low-density lipoprotein; SBP, systolic blood pressure; TG, triglycerides; WHR, waist-hip-ratio. Values for median (interquartile range) are shown. * indicates *p* < 0.05 as assessed by Mann–Whitney U test; ^#^ for FGF21 *n* = 39 controls and *n* = 30 LD were analyzed.

**Table 2:** Univariate correlations with GDF15 in the entire study population and multivariate regression analysis between GDF15 (lg; dependent variable) and age, group, gender, WHR (lg), SBP (lg), HbA1c (lg), HDL cholesterol (lg), LDL cholesterol, TG (lg), eGFR (lg), as well as CRP (lg). Non-normally distributed variables were logarithmically transformed (lg) prior to multivariate testing. r- and *p*-values, as well as standardized β-coefficients and *p*-values, are given, respectively. Abbreviations are indicated in Table 1. * indicates significant correlation as assessed by Spearman’s correlation method. ^†^ indicates significant correlation in multivariate analysis.

Parameter	Univariate Correlations	Multivariate Regression Analysis	
	r/p	β	*p*
Age (years)	0.494/<0.001 *	0.177	0.043 ^†^
Group (LD vs. Non-LD)	-	0.228	0.017 ^†^
Gender	-	0.005	0.943
BMI (kg/m^2^)	0.275/0.002 *	-	-
WHR	0.573/<0.001 *	0.079	0.404
SBP (mmHg)	0.265/0.003 *	-0.099	0.143
DBP (mmHg)	0.153/0.095	-	-
HbA1c (%)	0.588/<0.001 *	-	-
HbA1c (mmol/mol)	0.599/<0.001 *	0.225	0.005 ^†^
FG (mmol/L)	0.381/<0.001 *	-	-
FI (pmol/L)	0.389/<0.001 *	-	-
HOMA-IR	0.451/<0.001 *	-	-
Cholesterol (mmol/L)	0.003/0.974	-	-
HDL cholesterol (mmol/L)	−0.408/<0.001 *	0.085	0.450
LDL cholesterol (mmol/L)	−0.392/<0.001 *		
TG (mmol/L)	0.572/<0.001 *	0.323	0.004 ^†^
FFA (mmol/L)	0.179/0.056	-	-
Creatinine (µmol/L)	0.034/0.715	-	-
eGFR (mL/min/1.73 m^2^)	−0.277/0.002 *	−0.345	<0.001 ^†^
CRP (mg/L)	0.291/0.001 *	0.152	0.023 ^†^
Adiponectin (mg/L)	−0.263/0.004 *	-	-
Leptin (µg/L)	−0.198/0.030 *	-	-
FGF21 (pg/mL)	0.560/<0.001 *	-	-
Smoking	0.245/0.007 *	-	-
Metformin	0.493/<0.001 *	-	-

**Table 3:** Baseline characteristics, as well as parameters before and 6 months after initiation of metreleptin treatment in LD patients (*n* = 16). Abbreviations are indicated in Table 1. Values for median (interquartile range) or absolute numbers (*n*) are shown. * indicates *p* < 0.05 as assessed by Wilcoxon signed rank test.

Parameter	Baseline Characteristics		
*n*	16		
Age (years)	42 (18)		
Gender (male/female)	3/13		
	**Before treatment**	**6 months treatment**	***p***
GDF15 (ng/L)	1312.0 (1277.1)	1157.6 (888.0)	0.715
BMI (kg/m^2^)	27.4 (5.6)	27.3 (7.1)	0.035 *
WHR	0.97 (0.11)	0.96 (0.09)	0.331
SBP (mmHg)	128 (16)	128 (9)	0.754
DBP (mmHg)	80 (16)	74 (14)	0.510
HbA1c (%)	8.0 (2.2)	7.2 (1.3)	0.081
HbA1c (mmol/mol)	63.9 (23.8)	55.3 (14.5)	0.119
FG (mmol/L)	9.6 (2.9)	7.9 (4.2)	0.808
FI (pmol/L)	144.2 (280.0)	238.1 (374.4)	0.542
HOMA-IR	12.4 (11.0)	10.6 (22.2)	0.583
Cholesterol (mmol/L)	5.85 (4.94)	5.11 (4.32)	0.502
HDL cholesterol (mmol/L)	0.62 (0.48)	0.62 (0.42)	0.659
LDL cholesterol (mmol/L)	1.65 (2.09)	1.66 (1.79)	0.318
TG (mmol/L)	8.64 (14.78)	3.97 (6.18)	0.020 *
FFA (mmol/L)	0.70 (0.30)	0.63 (0.42)	0.594
Creatinine (µmol/L)	63 (20)	62 (25)	1.000
eGFR (mL/min/1.73 m^2^)	101.3 (41.1)	109.3 (33.4)	0.893
CRP (mg/L)	3.1 (5.4)	4.5 (4.5)	0.094
Adiponectin (mg/L)	2.2 (1.8)	2.0 (2.1)	0.382
Leptin (µg/L)	5.1 (4.6)	11.8 (20.6)	0.023 *
ALAT (µkat/L)	0.54 (0.46)	0.52 (0.33)	0.055
ASAT (µkat/L)	0.57 (0.57)	0.47 (0.52)	0.680
GGT (µkat/L)	1.04 (2.53)	0.91 (0.74)	0.194

**Table 4:** Baseline characteristics of the lipodystrophic animal setting (*n* = 8 per group).

Parameter	Control	Tg(SREBP-1c)		*p*
		Saline	Leptin	
*n*	8	8	8	
Age (years)				
Gender (male/female)	8/0	8/0	8/0	
BW (g)	23.3 (0.6)	22.1 (0.8) ^a^	17.8 (0.9) ^b^	0.0001 *
iBAT weight (mg)	57.5 (3.2)	193.8 (17.1) ^a^	121.1 (14.7) ^a;b^	<0.0001 *
sAT weight (mg)	348.4 (40.0)	80.2 (4.3) ^a^	53.1 (4.2) ^a^	<0.0001 *
epiAT weight (mg)	435.6 (30.3)	97.8 (6.0) ^a^	57.1 (6.0) ^a^	<0.0001 *
WHR	n.d.	n.d.	n.d.	
SBP (mmHg)	n.d.	n.d.	n.d.	
DBP (mmHg)	n.d.	n.d.	n.d.	
HbA1c (%)	n.d.	n.d.	n.d.	
HbA1c (mmol/mol)	n.d.	n.d.	n.d.	
FG (mmol/L)	6.3 (0.6)	7.8 (0.7)	7.7 (1.8)	0.526
FI (pmol/L)	n.d.	n.d.	n.d.	
HOMA-IR	n.d.	n.d.	n.d.	
Cholesterol (mmol/L)	31.1 (4.7)	42.7 (2.4)	35.0 (7.8)	0.325
HDL cholesterol (mmol/L)	n.d.	n.d.	n.d.	
LDL cholesterol (mmol/L)	n.d.	n.d.	n.d.	
TG (mmol/L)	2.0 (0.1)	7.4 (1.2) ^a^	3.5 (1.1) ^b^	0.0017 *
FFA (mmol/L)	0.5 (0.02)	0.7 (0.03) ^a^	0.6 (0.06)	0.0248 *
Creatinine (µmol/L)	n.d.	n.d.	n.d.	
eGFR (mL/min/1.73 m^2^)	n.d.	n.d.	n.d.	
CRP (mg/L)	n.d.	n.d.	n.d.	
Adiponectin (mg/L)	n.d.	n.d.	n.d.	
Leptin (µg/L)	n.d.	n.d.	n.d.	
FGF21 (pg/mL)	n.d.	n.d.	n.d.	
Smoking (*n*)	-	-	-	
Metformin (*n*)	-	-	-	
ALAT (µkat/L)	0.9 (0.13)	6.0 (0.69) ^a^	2.6 (0.58) ^b^	<0.0001 *
ASAT (µkat/L)	3.5 (0.6)	8.1 (0.9) ^a^	4.5 (0.8) ^b^	0.0013 *

ALAT, alanine aminotransferase; ASAT, aspartate aminotransferase; epiAT, epididymal adipose tisse; sAT, subcutaneous adipose tissue; iBAT, intrascapular brown adipose tissue; BW, body weight; CRP, C reactive protein; DBP, diastolic blood pressure; eGFR, estimated glomerular filtration rate; FFA, free fatty acids; FG, fasting glucose; FGF21, fibroblast growth factor 21; FI, fasting insulin; GGT, gamma-glutamyltransferase; HbA1c, glycosylated hemoglobin A1c; HDL, high-density lipoprotein; HOMA-IR, homeostasis model assessment of insulin resistance; LD, lipodystrophy; LDL, low-density lipoprotein; SBP, systolic blood pressure; TG, triglycerides. Values for mean (standard error of the mean) are shown. * indicates *p* < 0.05 as assessed by one way ANOVA; a;b indicates *p* < 0.05 corrected by Bonferroni–Holm as compared to a control and b saline-treated Tg(SREBP-1c).

## Abbreviations

ALAT	alanine aminotransferase
ASAT	aspartate aminotransferase
iBAT	intrascapular brown adipose tissue
BMI	body mass index
CRP	C reactive protein
eGFR, EpiAT	estimated glomerular filtration rate
FFA	free fatty acids
FG	fasting glucose
FGF	fibroblast growth factor
FI	fasting insulin
GGT	gamma-glutamyl transferase
H bA1c	glycosylated hemoglobin A1c
HDL	high density lipoprotein
HIV	human immunodeficiency virus
HOMA-IR	homeostasis model assessment of insulin resistance
LD	lipodystrophy
LDL	low density lipoprotein
sAT	subcutaneous adipose tissue
TG	triglycerides
VAT	visceral adipose tissue
WHR	waist-to-hip ratio
